# Establishing an expert opinion framework for lung volume reduction in Ireland: a Delphi consensus technique

**DOI:** 10.1007/s11845-023-03467-7

**Published:** 2023-08-08

**Authors:** Kathryn Mulryan, Jan Sorensen, Karen Redmond

**Affiliations:** 1 Professor Eoin O’Malley National Thoracic and Transplant Centre, Mater Misericordiae University Hospital, Eccles St., Dublin, D07R2WY Ireland; 2 Beacon Court, Beacon Hospital, Bracken Rd, Sandyford Business Park, Dublin, Sandyford Ireland; 3 School of Postgraduate Studies, Royal College of Surgeons in Ireland, Dublin, Ireland; 4 Healthcare Outcomes Research Centre, School of Population Health, RCSI University of Medicine and Health Sciences, Dublin, Ireland; 5 School of Medicine, University College Dublin, Dublin, Ireland

**Keywords:** Chronic obstructive pulmonary disease care, Consensus, Delphi technique, Practice guidelines

## Abstract

**Background:**

Lung volume reduction (LVR) is an effective treatment option offered to patients with emphysema. There is no formalised LVR referral network in Ireland. A rigorous approach to agreeing and implementing a LVR referral framework in an Irish context is required. A Delphi process was used to provide a basis for a framework of multi-disciplinary teams (MDTs) which can provide LVR as a management option. A Delphi process offers a framework for understanding variations and developing a consensus from expert opinion.

**Aim:**

The aim of this study was to develop consensus on recommendations for LVR referral guidelines in an Irish context and provide a national scope based on current practice and evidence.

**Design:**

In accordance with Guidance on Conducting and Reporting Delphi Studies, a consensus-building Delphi study was performed. Thirty-three statements informed from review of research literature were identified and presented to participants. Evaluation of the statements was performed by an expert panel using a 5-point Likert scale. ≥ 70% agreement was defined as consensus and items with a consensus rating of < 70% were revised during the process. In total, Delphi questionnaires were distributed to 18 experts with a response rate of 78% (*n* = 14) and a follow-up response-rate of 50% (*n* = 7).

Setting/participants.

The expert panel in Ireland consisted of representatives from respiratory medicine, cardiothoracic surgery and allied-health professionals with expertise in COPD care.

**Results:**

Of the initial 33 statements in five dimensions, there were consensus regarding 31 statements.

**Conclusions:**

The 31 statements agreed through this Delphi study clarify a coherent direction for development of a LVR framework in Ireland. The Delphi study methodology described is a useful process to reach consensus among multi-disciplinary experts.

## Introduction

Chronic obstructive pulmonary disease (COPD) is a leading cause of morbidity and mortality worldwide [1]. The HSE’s National Healthcare Quality Reporting System Reports (NHQRS) Annual Report 2019 estimates that almost 500,000 Irish people aged 40 years and over have COPD. Over 200,000 have moderate or severe disease and only half are likely to be diagnosed. As the population increase and age, these numbers are likely to increase [2, 3]. The ‘End to end COPD model of care’, a guideline for the treatment of COPD in Ireland was recently published [2]. This document provides a roadmap for patients and their healthcare providers for the overall care and treatment of patients with COPD. Smoking cessation, pulmonary rehabilitation, and optimisation of nutrition, oxygen prescription, medication and surgical procedures are interventions in the treatment of COPD. There is scope to build upon this document with the provision of a multi-disciplinary team (MDT) which is linked to access to surgical procedures for patients with COPD; namely lung volume reduction surgery.

Lung volume reduction (LVR) is a treatment option offered to patients with emphysema, where areas of diseased lung are resected to reduce hyperinflation. The National Emphysema Treatment Trial (NETT) demonstrated the clinical benefit of LVR in selected subgroups [4]. Robotic assisted LVR has recently been introduced into clinical practice as an alternative to thorascopic LVR [5]. Recently, the use of endoscopic endobronchial valves has demonstrated improvements in pulmonary function, exercise capacity, and quality of life [6]. Lung transplantation is a last resort for these patients and involves removing the entire hyperinflated lung. This procedure is subject to limited organ availability and has restrictive eligibility criteria.

LVR has been recently commissioned in England, where a multi-disciplinary team approach is recommended to decide the appropriate intervention for a patient with COPD [7]. Previous MDTs, known as emphysema MDTs or breathlessness MDTs, had been set up in England to allow for the identification and referral of the correct patient population and to increase access to procedures that can improve quality of life [8]. One study found that patients presented at a dedicated MDT had increased access to LVR, presented for less re-interventions and had better breathing post intervention [8]. LVR is not routinely offered in an Irish setting, with no agreed referral pathway and no formalised funded MDT. This research aims to provide clarification regarding an MDT approach linked to offering LVR from an expert group of stakeholders using a Delphi process. There is a precedence to establish a network; with the National Cancer Control Programme funding lung cancer services (8 regional rapid access clinics with key performance indicator (KPI)-regulated MDTs and additional supports to 4 thoracic surgery units) in 2012 [9].

Delphi techniques are used internationally to investigate a wide variety of issues. The purpose is to develop an expert-based judgement derived from consensus agreement. One aspect is that experts included in a Delphi process can use personal expertise, knowledge from literature including randomised-controlled trials and knowledge from other participants in the process [10]. It is useful in the generation of policy solutions using an iterative process under uncertain conditions [11]. A panel of experts (unknown to each other) is created to participate in two or more questionnaire rounds. A moderator collates results from the first round and resubmits a questionnaire for participants to re-consider. The defining feature of a Delphi review is that feedback is given to the participants from the initial round and opportunity is given to revise or reconsider their previous judgement, revising if appropriate. Once consensus has been achieved, there are no further rounds. The Delphi technique involves both qualitative and quantitative elements.

## Method and materials

### Ethical approval was granted under Beacon Hospital Institutional Review Board (Ref: BEA0153)

This study involved two rounds of a self-administered web-based survey which was distributed using Googledocs™. The authors identified existing evidence gaps in the co-ordination of LVRS services in Ireland, with no formalised MDT or allocated provision of service by the Health Service Executive (HSE). The topics under evaluation included the referral criteria for a screening outpatient clinic, patient pathway for LVR, COPD screening clinic process, COPD MDT members and the national framework of the MDT. In preparation for each round, piloting of the survey was performed by trainee doctors on medical training schemes with no competing interests in this area of research to ensure readability, identify errors, improve efficiency in administration and remove any ambiguity in questions. The time needed to complete the survey was estimated to be approximately 20 to 30 min.

The questionnaire evaluated this using both quantitative and qualitative means. It comprised of states to which the respondents could indicate agreement/disagreement using the 5-point Likert scale, which is a 1- to 5-point psychometric response scale. Respondents specify their level of agreement to a statement typically in five points:

1. Strongly disagree
2. Disagree
3. Neither agree nor disagree
4. Agree
5. Strongly agree

Expert stakeholders were identified and invited to participate via email. These anonymous stakeholders were chosen based on their contribution to COPD care and development of COPD or respiratory services in Ireland. It included a wide spectrum of representatives from specialities involved in the management of LVRS patients and who could be involved in an MDT process. This included physicians, surgeons, and allied health members.

Participant comments were sought at the end of each topic. These comments were provided in the subsequent iterations of the questionnaire. Validity and reliability of the survey as related to LVR were evaluated through expert opinion. References and literature around each topic were provided in a Dropbox™ folder accessible to participants.

### Round one

Invitations to the round one survey were sent to invited participants, with the survey remaining open for 2 weeks. One follow-up reminder email was sent prior to the closure of the survey.

Statements were grouped into five dimensions:

- Referral criteria for a screening clinic
- Patient pathway for LVR
- COPD screening clinic
- COPD MDT members
- The National framework of the LVR MDT

There is no set definition of consensus in a Delphi Consensus Study; however, reviews indicate that commonly used definitions for consensus range between 51 and 80% [12]. A threshold of 70% was used in this study to define consensus.

### Round two

Invitations to participate in round two were sent to participants who had answered the initial round. Results of the 33 statements originally presented in round one were analysed and returned to the participants where their opinion was sought on items *that had not achieved consensus* in the first round. Anonymous comments from round one from participants were provided to participants to allow group discussion and revision of opinions in gaining a group consensus. After the second round, results were collated and again re-distributed as consensus had not been achieved. The survey remained open for 2 weeks and one reminder was sent prior to the survey closing. Round two included only those statements that did not achieve consensus in round one. In round two, the panel used the same voting method as described for round one. If consensus had not been achieved in round two, statements would again be revised and re-distributed for discussion.

### Delphi process: data analysis

Fully completed questionnaires were included for data analysis. Basic descriptive statistics are calculated to establish the panellists’ consensus. The percentage of respondents in agreement (i.e. those who chose ‘Agree — 4’ or ‘Strongly agree — 5’ on the Likert scale) is used to determine level of consensus.

## Results

### Participants

Eighteen respiratory consultants, thoracic surgeons and other MDT members including representatives from allied healthcare were invited to participate in Round one. All participants had expertise in the management of patients with COPD, and work within the Irish or UK healthcare systems. Fourteen responses were received from Round one (78%; *n* = 14/18). Seven members participated in the second round. Panellist anonymity was maintained throughout the process. All comments were incorporated anonymously in the statements and questionnaires distributed to panellists in each round.

### Round one

**Fig. 1:**
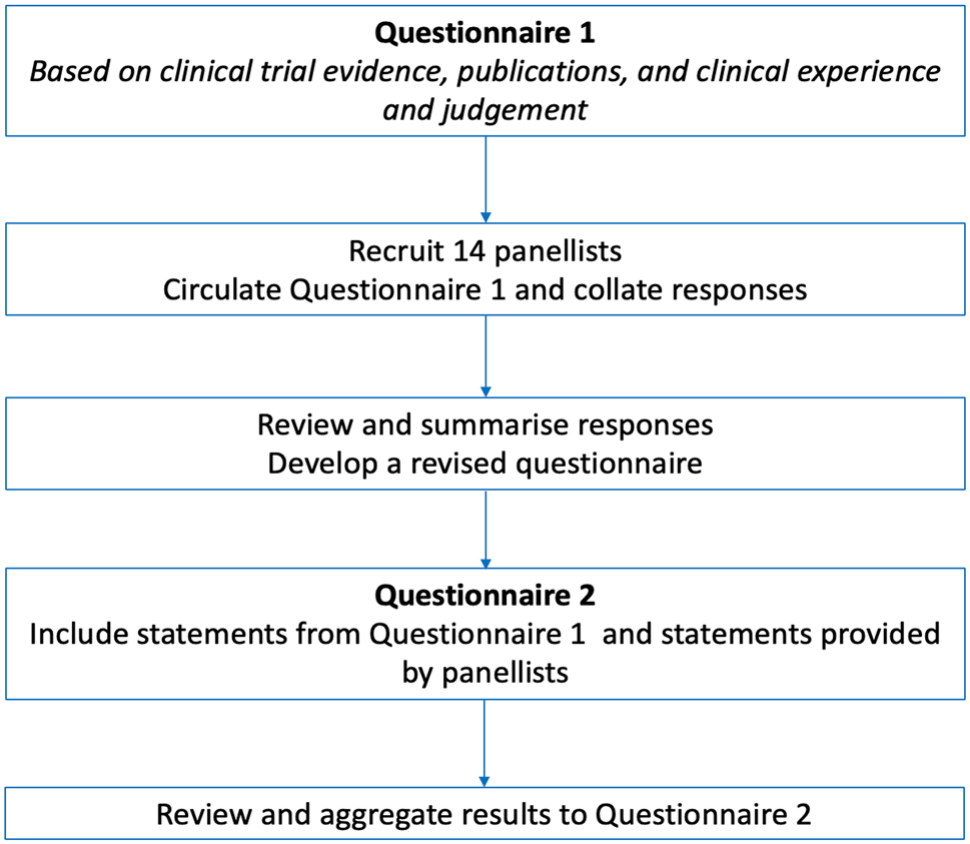
Delphi process flow chart

In round one, 33 statements were presented. Of these; seven pertained to referral criteria; six suggested guidelines for investigations to be completed prior to referral; two questions were asked regarding the assessment of patients in a specialist COPD clinic; twelve statements were provided to ascertain MDT membership and six criteria were given to provide a guideline for the national introduction of LVR in Ireland. Figure 1 and Table 1 illustrates the flow of the results of the Delphi method.

**Table 1:** Statements that have reached consensus after the Delphi process are outlined below: Referral Criteria

	**Round one** **(% consensus)**	**Round two** **(% consensus)**
Patients with FEV1 (forced expiratory volume in 1 s) < 50% should be considered for LVR assessment once non-surgical management has been optimised	*	100%
Patient with a residual volume of > 150% (Body-Box) should be considered for LVR assessment	78.6%	-
Patients with evidence of radiological hyperinflation impacting on activities of daily living (ADLs) should be considered for LVR assessment	78.6%	-
The 6-min walk test (6MWT) should not be used as a mandatory referral criterion	*	71.5%
Patients should already attend (or have attended in the past 12 months) a pulmonary rehabilitation group in order to be considered for LVR assessment	78.6%	-
Patients should be a non-smoker for at least 3 months to be considered for LVR assessment	85.7%	-
Supplementary oxygen requirements should not be used to exclude referral of patients with COPD for LVR	*	85.7%

### Round two

In round two, participants were provided with the results of the previous round, including the criteria that had reached consensus. Several statements were reworded based on the commentary given in the first round. A total of twelve statements were considered by the expert panel in the second round.

A total of thirty-one statements achieved consensus and are outlined below.

## Discussion

This study was conducted to facilitate a formal consensus being reached on guidelines for the appropriate referral for surgical management of patients with COPD. Using a Delphi approach, this research reached consensus on a total of 30 statements. These are the first statements that have been specifically formulated to provide guidelines on the management of patients with COPD in an Irish setting. It was developed through the participation of Irish clinicians and external experts in LVRS in the UK.

Guidelines have been recently introduced for the NHS (National Health Service, England) for the implementation of LVR. ADDIN EN.CITE Clinical Commissioning Policy Proposition: Lung volume reduction by surgery or endobronchial valve for severe emphysema in adults [7] Clinical Commissioning Policy Proposition: Lung volume reduction by surgery or endobronchial valve for severe emphysema in adults NHS England Reference: 200806P [1622] 2 England, N.H.S 1644340270 46 2022 2011 NHS England 1,644,340,609 National Institute for Health and Excellence (NICE) Commissioning Support Programme [7] Referral criteria are largely similar between our results and those recommended by the National Institute for Health and Care Excellence (NICE); namely to “…have severe COPD, with FEV1 less than 50% and breathlessness that affects their quality of life despite optimal medical treatment and do not smoke and have completed pulmonary rehabilitation and do not have contraindications for transplantation (for example, comorbidities or frailty)” [7]. Notably, the panellists in this consensus review do not recommend a minimum 6MWT distance to allow referral for consideration of LVR. This is in contrast to a 6MWT of at least 140 m recommended by NICE [7]. Expert commentary provided in this section found in their clinical experience that the “*walk test gives us the most problems and if a patient satisfies all other criteria […] we continue as long as they are keen to have it and look reasonable otherwise*”. Experts commented that the pulmonary rehab referral criterion may be overly stringent, however this did achieve consensus in round one with 78.6% agreement. *“Huge variation in access to PR programmes nationwide mean some patients excluded from consideration due to postcode lottery” “Limited access to or availability of pulmonary rehabilitation should not preclude someone from LVR procedures”* Limited access to pulmonary rehabilitation is currently being addressed as per the National Clinical Programme Respiratory End-to-End Model of Care for COPD recommendations [2] with the introduction of 34 community-based dedicated pulmonary rehabilitation teams [13].

**Fig. 2:**
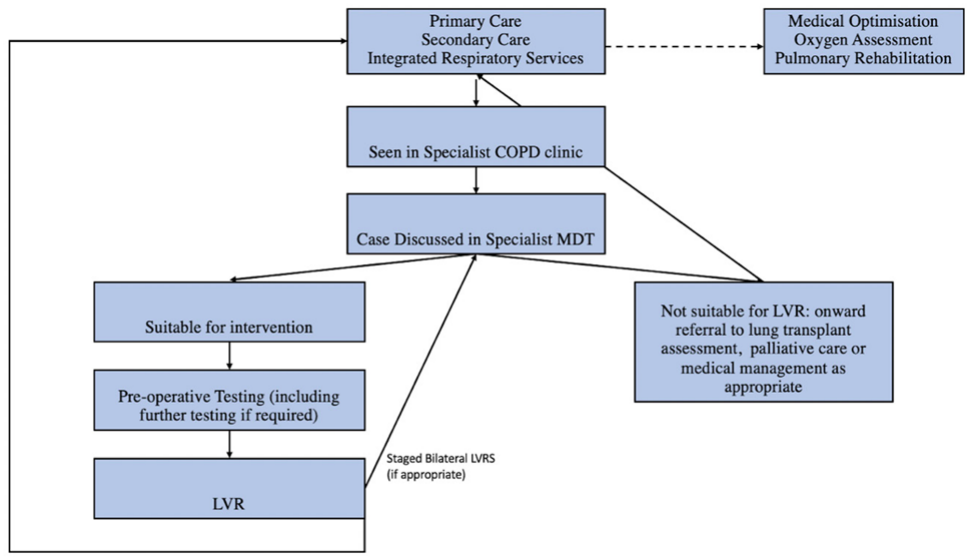
Proposed Patient Referral Pathway for Surgical Management of COPD provided to panellists

The patient pathway as outlined in Fig. 2 reached consensus in Round One (78.6%). Consensus was similarly reached on investigations required prior to referral; namely PFTs, with capture of residual volumes, CT thorax and 6MWT. These are core investigations which define the eligibility for patients to undergo any LVR procedure. A requirement for patients to undergo arterial blood gas (ABG) testing did not reach consensus in the first round with expert’s commenting that *“blood gases are not routinely done if [oxygen saturations are] reasonable in our pathway”*. An ABG pre-referral would identify pre-existing supplementary oxygen requirements, recommended for patients with pO_2_ > 8 kPa. As 6MWTs are carried out prior to any LVR procedure, it is anticipated that patients with this requirement would be diagnosed later in the pathway, during pre-operative investigations (Fig. 2).

Core members of the COPD MDT, as agreed upon by group consensus are a respiratory consultant physician, interventional bronchoscopist, consultant pulmonary radiologist, lung transplant consultant, thoracic surgeon, respiratory nurse and a physiotherapist. Members agreed to be part of the extended, non-mandatory team include occupational therapist, palliative care consultant physician, social worker, clinical psychologist and exercise physiologists. The inclusion of these members, while holding an integral role in the care of patients with COPD, did not reach consensus in the first round. *“[…] essential members should include Respiratory Physician (specialising in COPD), Interventional Bronchoscopist, Thoracic Surgeon, Lung Transplant consultant, Consultant Pulmonary Radiologist, and Respiratory nurse. Desired members Palliative care consultant, Psychologist and Physiotherapist”* Another commentator stated that *“[…] a pathway should exist back to the patient's Integrated Respiratory Service should surgery not be the ultimate outcome. An Integrated Respiratory Service should already have the required pathways/links with Palliative Care/Elderly Medicine/Psychology/Psychiatry/Local Chronic Disease Management supports *etc*.”* After considering the general expert commentary, and results of the initial round, we proposed that the members whose inclusion did not reach consensus could be viewed as auxiliary members, who may be invited to join MDT as per the needs of the local institution and consensus was reached in round two (Table 2). The ‘core’ team members as designated by consensus are similar to literature published from emphysema or hyper-inflation MDTs in the UK [14, 15].

**Table 2:** Proposed Patient Referral Pathway for Surgical Management of COPD provided to panellists

	**Round one** **(% consensus)**	**Round two** **(% consensus)**
Do you agree with the above proposed pathway as outlined in Fig. 2?	78.6%	-
All patients meeting specified criteria for LVRS should be referred to a specialist end-stage COPD outpatient clinic	78.6%	-
Minimum investigations required before discussion at an LVRS MDT should include: pulmonary function tests (PFTs) with Body Box plethysmography	92.8%	-
Minimum investigations required before discussion at an LVRS MDT should include: computed tomography (CT) thorax non contrast, high resolution	92.9%	-
Minimum investigations required before discussion at an LVRS MDT should include: six minute walk test	78.5%	-
An ABG to diagnose oxygen requirements before referral for LVR assessment is not a required criterion but should be included if available	*	85.7%

COPD/LVRS screening clinic		
Review of referred patients with COPD for consideration of LVRS should be in a consultant led OPD supported by an advanced practitioner physiotherapist and/or a COPD advanced nurse practitioner	*	100%

COPD MDT membership		
Respiratory Consultant should be a member of a COPD MDT	100%	-
Interventional Bronchoscopist / Respiratory Consultant should be a member of a COPD MDT	78.6%	-
Consultant Pulmonary Radiologist should be a member of a COPD MDT	92.9%	-

COPD MDT membership		
Respiratory Consultant should be a member of a COPD MDT	100%	-
Interventional Bronchoscopist / Respiratory Consultant should be a member of a COPD MDT	78.6%	-
Consultant Pulmonary Radiologist should be a member of a COPD MDT	92.9%	-
Lung transplant consultant should be a member of a COPD MDT	78.6%	-
Thoracic Surgeon should be a member of a COPD MDT	92.8%	-
Respiratory Nurse should be a member of a COPD MDT	92.8%	-
An occupational therapist should be a member of the extended (non-mandatory) COPD MDT	*	85.7%
A palliative care consultant should be a member of the extended (non-mandatory) COPD MDT	*	85.7%
Social workers should be a member of the extended (non-mandatory) COPD MDT	*	100%
Exercise physiologist should be a member of the extended (non-mandatory) COPD MDT	*	85.7%
Physiotherapist should be a member of a COPD MDT	85.8%	-
Clinical Psychologist should be a member of the extended (non-mandatory) COPD MDT	*	85.7%

National scale		
COPD MDTs should be affiliated with pulmonary rehabilitation services already in place	92.9%	-
COPD MDTs should be affiliated with oxygen therapy services already in place	92.9%	-
LVRS should be discussed at MDT prior to procedure	*	100%
LVR should be considered in a hospital setting where an interventional bronchoscopist and/or a thoracic surgeon are available and have access to the full MDT	*	100%

*These statements were introduced in Round two and were modifications of statements, based on expert commentary, that did not reach consensus in Round one

An issue which is raised repeatedly in literature and in many of the expert commentators is that access to LVR can be a ‘postcode lottery’. This can also be seen throughout the provision of services to patients with COPD in Ireland. Limited access to pulmonary rehabilitation and assessment for long-term oxygen therapy was mentioned by several experts in the Delphi Consensus. These necessary services should be linked to primary and secondary level care of patients with COPD, as achieved agreed upon by members of the consensus (Table 2). Similarly, LVR remains a postcode lottery with no dedicated MDT linked referral network in Ireland. One reason for a lack of national access to LVR could be the cost associated with these services. The costs associated with LVR needs to be defined in the context of their well-known improvement in QoL parameters [16].

## Conclusion

Overall, this was a robust methodology for achieving a rigorous consensus within this multidisciplinary group of experts. Consensus has been achieved with 31 statements pertaining to an LVR MDT on a national scale. A set of guidelines have been formulated from this group accord.

## Data Availability

Any data not included in this paper can be provided, on request.
